# Supplementary material for: WordCommentsAnalyzer: A windows software tool for qualitative research
Source: F1000Res. 2018 Sep 4;7:536. Originally published 2018 May 3. [Version 2] doi: 10.12688/f1000research.14819.2 (PMC6137411; doi:10.12688/f1000research.14819.2)
Supplement: Supplementary file 3 [file f1000research-7-17614-s0002.tgz › b2a79496-4ada-47c4-9682-a99ce867836d.zip!/Eight Iranian Health Journals/Journal of Health System Research _24.docx]

© Journal of Health System Research . This work is licensed under CC BY-NC 4.0 (https://creativecommons.org/licenses/by-nc/4.0/).

Volume & Issue: دوره 9، شماره 8، 1392

Title: مروری بر سازه‌های سلامت اجتماعی در پرتو کارکردهای آشکار و پنهان نماز؛ یک مطالعه مروری

http://hsr.mui.ac.ir/index.php/jhsr/article/view/941

نماز، بزرگ‌ترين عبادت و مهم‌ترينسفارش انبياء الهی به ویژه پیامبر اعظم است. نماز، جزء جدايى‌ناپذير همه شرايعالهى بوده و در هر دينى به صورتى خاص وجود داشته است. نمازدر اسلام و قرآن از اهمیت و منزلت خاصی برخوردار است که آثار و برکات این فرضیهبزرگ در جای جای کتاب آسمانی با تعابیری لطیف و نغز و موجز بیان شده به گونه‌ای کهتعمق در آنها شور و جزبه‌ای وصف‌ناپذیر در پویندگان طریق سعادت بر می‌انگیزد. فلسفه،فايده و آثار، شرايط و آداب و اوصاف نماز در آيات و روايات بسياری آمده كه پرداختنبه همه آنها در اين جا ممكن نيست. از سویی مقوله سلامت اجتماعیو تحقق آن در جوامع انسانی ضرورتی اجتناب‌ناپذیر است. بشر تلاش‌های زیادی باسرمایه‌گذاری‌های خرد و کلان و استفاده از مدرن‌ترین امکانات جهت دستیابی بهآسایش، امنیت، حیات و سلامت اجتماعی طی قرون متمادی انجام داده اما وقایع اجتماعیو آمارهای موجود در این خصوص مبین این امر است که انسان در این راستا به توفیقاتچشمگیری نرسیده و سلامت اجتماعی هنوز یکی از اساسی‌ترین دغدغه‌های بشر است. به هر حال مطالعه راجع بهسلامت اجتماعی، ابعاد آن و عوامل پیش‌بینی‌کننده و ارتقاء‌دهنده آن به دلیل اهمیتفراوان همیشه توجه جامعه‌شناسان و کارشناسان حوزه‌های اجتماعی سلامت را به خود جلبنموده است. اگر چه به ابعادسلامت جسمانی و روانی نماز در گذشته توجه شده و مطالعاتی صورت گرفته اما بعد سلامتاجتماعی نماز همچنان مغفول مانده و نیازمند بررسی و تحقیقات بیشتر می‌باشد.نویسندگان این مقاله سعی نموده‌اند تا در این بررسی، به سازه‌های سلامت اجتماعینماز بر اساس آیات، روایات و احادیث پرداخته و هر یک را به تفکیک معرفی و طبقه‌بندینمایند.

Title: تحلیل عاملی عوامل موثر بر بیمارستان متعهد به ارتقای سلامت در بیمارستان های منتخب شهر اصفهان

http://hsr.mui.ac.ir/index.php/jhsr/article/view/923

مقدمه: بهطور خلاصه یک سیستم بهداشتی، بدون وجود نظام بیمارستانی کارا و موثر نمی‌تواند به اهداف خود نایل گردد. برای نیل به این مسأله، به بیمارستان‌هایی نیاز است که به برنامه ارتقای سلامت به عنوان یک خدمت کلیدی مهم توجه داشته باشند. از این رو اینمقاله با هدف تعیین عوامل موثر بر بیمارستان متعهد به ارتقای سلامت صورت گرفته است.روش‌ها: اين تحقيق از نوع تحقيقات توصیفی –همبستگیاست. جامعه مورد پژوهش بيمارستان‌های منتخب شهر اصفهان می‌باشند. نمونه‌گیری به روشتصادفی طبقه‌ای صورت گرفته است. ابزار جمع‌آوري داده‌ها پرسشنامه محقق ساختهارتقای سلامت بوده که اعتبار و روايي آن مورد تاييد قرار گرفته است. برای تعیینعوامل از تحلیل عاملی اکتشافی استفاده شد .تعیین کفایت حجم نمونه با استفاده ازروش کیسر مایر الکین صورت گرفت. روش تحلیل داده‌ها، تحلیل عاملی و نرم‌افزار مورداستفاده SPSS بوده است.یافته‌ها: در بین مولفه‌های موثر نیازسنجی (ارزیابینیاز) ارتقای سلامت برای گروه‌های بیماری متفاوت (90/0)توانمندسازی کارکنان برای ارتقای سلامت (82/0)، تعریف الزامات ایمنی و سلامت درمحیط کار (84/0)، توانمندسازی جامعه برای مدیریت بیماری‌ها (88/0) دارای بالاترینمیزان تاثیر بوده‌اند.نتیجه‌گیری: باتوجه به میزان تاثیر مولفه‌ها،می‌توان نتیجه گرفت که بیمارستان‌ها برای استقرار برنامه‌های ارتقای سلامت، بایستیبه ارتقای سلامت جامعه وکارکنان توجه ویژه‌ای نماید.

Title: رضایتمندی مراجعین بخش اورژانس بیمارستان آموزشی الزهرا (س)

http://hsr.mui.ac.ir/index.php/jhsr/article/view/960

مقدمه: سطح رضایتمندی، ﯾﮏ ﺷﺎﺧﺺمهم در ﺳﻨﺠﺶ ﮐﯿﻔﯿﺖ ارایه ﺧﺪﻣﺎت است و ارﺗﻘﺎء ﮐﯿﻔﯿﺖ ﺧﺪﻣﺎت ارایه ﺷﺪه در ﺑﺨﺶ اورژاﻧﺲﻧﯿـﺎز ﺑـﻪ ﺷﻨﺎﺧﺖ ﺻﺤﯿﺢ وﺿﻌﯿﺖ ﻣﻮﺟﻮد و ﺑﺮرﺳﯽ ﻣﺸﮑﻼت اﯾﻦ ﺑﺨﺶ‌ﻫـﺎ دارد. مطالعه حاضر با هدف بررسی میزان رضایتمندی مراجعین از خدمات ارائه شده در بخشاورژانس بیمارستان آموزشی درمانی الزهرا (س) انجام گرفت.روش‌ها: اين مطالعه به صورت توصيفي– تحلیلی در سال 1390-1391 در بین 780 مراجعه‌کننده به بیمارستان آموزشی درمانیالزهرا شهر اصفهان انجام شد. ابزار پژوهش پرسشنامه محقق ساخته و روایی و پایاییشده بود. جهت دستيابي به نتايج و يافته‌ها، اطلاعات در برنامه نرم‌افزاري SPSSنسخه 17 درج و مورد تجزيه و تحليل قرار گرفت. یافته‌ها: سطح کلی رضایت 1/7٪ افراد ضعیف، 2/34٪ متوسط و 7/58٪ افراد در سطحخوب بود. بینابعاد رضایت و جنسیت، پوشش بیمه‌ای و نحوه انتقال - رابطه معنی‌داری به دست نیامد (05/0<P). تمامی ابعاد رضایت (به جز بعد رضایت از پرسنل غیردرمانیکه با تعداد دفعات مراجعه ارتباطی یافت نشد) با تحصیلات و تعداد مراجعه رابطه معنی‌داریداشتند (05/0>P). همچنین نتایج نشان داد با افزایش سن، میزان رضایت ازمدیریت بخش افزایش و رضایت از امکانات رفاهی و محیط فیزیکی کاهش می‌یابد.نتیجه‌گیری: در حالی که خدماتارائه شده به مراجعین اورژانس به طور نسبی رضایت بخش گزارش شد، در بعد امکاناترفاهی و فضای فیزیکی میزان نارضایتی بالا بود، بنابراین پیشنهاد می‌شود این بیمارستاندر جهت ایجاد فضای آرام و امکانات رفاهی برای بیمار و همراهان تلاش کند.

Title: بررسی روایی و پایایی ویرایش فارسی پرسشنامه جو ایمنی نوردیک (NOSACQ-50)

http://hsr.mui.ac.ir/index.php/jhsr/article/view/1069

مقدمه:جوایمنی توصيفی از بینش‌های مشترک کارکنان از نحوه‌ی مدیریت ایمنی بوده و ارزیابی آنيکی از ملزومات برنامه‌های ارتقا رفتارهای ايمن در محيط‌های کاری به شمار می‌رود.يکی از ابزارهای معتبر جهت ارزیابی جو ایمنی پرسشنامه جو ایمنی نوردیک (NOASACQ-50) می‌باشد که روايایی و پايايی آن در زبان‌های مختلفی مورد بررسیقرار گرفته است. هدف از اين مطالعه تهيه ويرايش فارسی پرسشنامه مذکور و سپس بررسیروایی و پایایی آن می‌باشد.روش‌ها: در مرحله اول روايیزبانی نسخه فارسی پرسشنامه از طريق ترجمه به زبان فارسی و بالعکس انجام شد و درمرحله بعد ويژگی‌های روان‌شناختی آن در بين 200 نفر از کارکنان يک صنعت کاشی وسراميک مورد ارزيابی قرار گرفت. برای سنجش پايايی پرسشنامه نيز از آزمون Kuder Richardson-20 استفاده شد. داده‌ها با استفاده از نرم‌افزارSPSS  نسخه19 تجزیه و تحلیل شد. یافته‌ها:دربررسی پايايی پرسشنامه با استفاده از روش ثبات داخلی، کمترين و بیشترين مقدار ضريبکودر ریچاردسون به ترتيب مربوط به بعد اولویت ایمنی کارکنان و ریسک‌ناپذیری(684/0) و بعدهای قدرت ایمنی مدیریت (926/0) و اولویت و توانایی ایمنی مدیریت(923/0) به دست آمد. ضريب کودر ریچاردسون برای کل حيطه‌های جو ایمنی مورد بررسی درپرسشنامه نيز 942/0 به دست آمد. نتیجه‌گیری: ویرایش فارسی پرسشنامهجو ایمنی نورديک از روایی و پايايی قابل قبولی برای ارزيابی جو ايمنی برخورداراست.

Title: تعيين راندمان بيوراكتور غشایي در تصفيه شيرابه حاصل از كارخانه كمپوست

http://hsr.mui.ac.ir/index.php/jhsr/article/view/1195

مقدمه: شيرابه داراي مقادير بالايي از آلودگي‌هايآلي به همراه آمونياك، هيدروكربن‌هاي هالوژنه، فلزات سنگين و نمك‌هاي غيرآلي است. ازمیان روش‌های مختلف، فرايندهاي غشایي براي تصفيه شيرابه همراه يك فرايند بيولوژيكيبيشتر توسعه پيدا كرده است به طوري كه از فرايندهاي غشایي براي جداسازي مايع- جامداستفاده مي‌گردد. هدف از این مطالعه،تعيين راندمان بيوراكتور غشایي در تصفيهشيرابه حاصل از كارخانه كمپوست است.روش‌ها: مطالعه حاضر يک مطالعه تجربی است کهبه صورت مقطعي در مقياس آزمايشگاهي انجام شد.در این مطالعه راکتور SBR 5 لیتری درتلفیق با غشای فیبر توخالی با تخلخل حدود 50٪ و فشار MPa 02/ 0بهره‌برداری می‌شد. حدود150 نمونه از نظر شیمیایی آنالیز شد که ميانگين غلظت COD، آمونياك، فسفرTSS شيرابه ورودي و خروجي راكتورMBR و نیز راندمان حذف پارامترهایمذکور توسط فرایند مورد بررسی قرار گرفت.یافته‌ها: راندمانبیوراکتور در حذف COD، BOD5،TSS ،آمونياك و فسفربه ترتیب حدود 2/99، 99،2/98،87 و 98٪ بوده است. طبقآزمون tزوجی تفاوت معنی‌داری از نظر آماری بین مقادیر ورودی و خروجی بیوراکتور غشاییوجود دارد(05/ 0P-value<). این امر نشانگر راندمان مناسب بیوراکتوردر حذف آلاینده‌های شیرابه بوده است. ولی بر اساس آزمون tتک نمونه‌ای، مقادیر پارامترهای کیفی پساب خروجی از بیوراکتور با مقادیراستاندارد سازمان محیط زیست تفاوت معنی‌داری دارد (05/ 0P-value<).نتیجه‌گیری: بر خلاف راندمان بالای بیوراکتور، برایدستیابی به خروجی مطابق با استانداردهای محیط زیست، انجام پیش تصفیه شیرابه خام بافرایندهایی نظیر میکرواسکرینینگ، ترسیب شیمیایی، عریان‌سازی بار بالای آمونیاک،راکتورهای بی‌هوازی بسیار مؤثر خواهد بود.

Title: سواد سلامت و رفتارهای ارتقادهنده سلامت در سالمندان شهر اصفهان

http://hsr.mui.ac.ir/index.php/jhsr/article/view/1250

مقدمه: سواد سلامت عبارت از میزان ظرفیت فرد برای کسب، تفسیر، درک اطلاعات اولیه وخدمات سلامتی است که برای تصمیم‌گیری متناسب لازم می‌باشد. سالمندان به عنوان یکیاز مهمترین گروه‌های در معرض خطر اثرات ناخوشآیند سطح پایین سواد سلامت به شمار می‌آیند.رفتارهاي ارتقادهنده سلامت در سالمندان، تاثير بالقوه‌اي در پيشبرد سلامتي و كيفيت زندگي آنان داشته و به همان نسبت موجب كاهش هزينه‌هاي مربوط به مراقبت‌هاي بهداشتي خواهد شد. این مطالعه با هدف تعیین ارتباط سواد سلامت و رفتارهای ارتقادهنده سلامتی درسالمندان شهر اصفهان به عنوان یک گروه آسیب پذیر انجام شد.روش‌ها: این مطالعه توصیفی- تحلیلی و به شیوه مقطعی بر روی 354 نفر سالمند که با روشنمونه‌گیری خوشه‌ای متناسب با حجم جامعه انجام شد. ابزار گردآوری داده‌ها پرسشنامهسواد سلامت عملکردی بزرگسالان (TOFHLA) و سیاههوارسی مربوط به رفتارهای ارتقادهنده سلامت بود. اطلاعات به روش مصاحبه و به صورت پرسشگریدرب منازل جمع آوری شدند. برای تحلیل داده‌ها از نرم افزار SPSS ویرایش 18، آمارتوصیفی و آزمون‌های آنالیز واریانسیک طرفه و کای اسکوئر استفاده شد.یافته‌ها: میانگین سنی سالمندان 97/6±67 بود. در این مطالعه بینسطح سواد سلامت و انجام فعالیت فیزیکی و مصرف میوه و سبزی ارتباط آماری معناداری مشاهده شد (001/0 P<). به طوری که در سالمندان با سواد سلامت بالاتر میزانفعالیت فیزیکی و مصرف روزانه میوه و سبزی بیشتر بود. بین سطح سواد سلامت و وضعیتسیگار کشیدن ارتباط آماری معناداری مشاهده نشد.نتیجه‌گیری: با توجه به نتايج پژوهش برنامه‌ريزي در جهت آموزش و توسعه رفتارهاي ارتقادهنده سلامت در سالمندان و لزوم توجهبیشتر به امر سواد سلامت در برنامه‌های ارتقا سلامت توصیه می‌گردد.

Title: بررسی کارایی فرایند کمپوست سازی در حذف باکتری‌های شاخص و پاتوژن از مواد زاید شهری در کارخانه کمپوست شهر اصفهان

http://hsr.mui.ac.ir/index.php/jhsr/article/view/1271

مقدمه: برای تولید کمپوست مناسب، پایش میکروارگانیسم‌های شاخص و پاتوژن ضروری است. این مطالعه با هدف بررسی باکتری‌های کلیفرم و سالمونلا  در کمپوست‌سازی از زایدات شهری و پس از استفاده ازکمپوست بر روی خاک، انجام شد. روش‌ها: نمونه‌برداری از 5 قسمت شامل زباله خام، توده اولیه، مرحله فعال، خنک شدن و محصول مرحله فعال (ترموفیلیک)، خنک شدن و محصول (تثبیت)، در ماه‌های آذر، دی و بهمن 1390 به دفعات 5 مرتبه انجام شد. پارامترهای  بررسی شده عبارتند از: دما، رطوبت، C/N و pH ، کلیفرم‌های کل و مدفوعی و سالمونلا . برای بررسی وضعیت کمپوست در خاک، 5 کیلوگرم از کمپوست بر روی خاک پخش گردید و تا 20 روز پس از استفاده، نمونه‏برداری و آزمایشات فیزیکی، شیمیایی و بیولوژیک در فواصل 5 روز یکبار انجام شد. یافته‌ها: حداقل و حداکثر دما مربوط به زباله خام و مرحله ترموفیلیک با C° 22 و C°61 بود. میانگین کلیفرم‌های کل و مدفوعی از MPN/gDW107×71/1 و 107×02/1 در زباله خام به MPN/gDW 103×3/2 و 103×18/1 در محصول کمپوست کاهش یافت. میانگین سالمونلا  در زباله خام MPN/gDW 415 بوده که مقدار آن به MPN/gDW28 در کمپوست رسید. جمعیت کلیفرم‌های کل و مدفوعی پس از استفاده از کمپوست روند کاهشی داشته و پس از 20 روز به حدود MPN/gDW 150 و 18 رسید. مقدار سالمونلا  نیز از MPN/gDW 28 در کمپوست در طول 5 تا 10 روز به نزدیک صفر رسید. نتیجه‌گیری: این بررسی نشان داد مرحله ترموفیلیک بیشترین تاثیر را بر روی کاهش باکتری‌های کلیفرم و مرحله تثبیت بیشترین تاثیر را بر سالمونلا  دارد. اما با توجه به آلودگی کمپوست استفاده شده بر روی زمین به دلیل بالاتر بودن مقادیر کلیفرم مدفوعی و سالمونلا  نسبت به مقادیر استاندارد، بایستی برای کاربرد این کمپوست جهت محصولاتی که در تماس با زمین هستند فاصله زمانی مناسب بین استفاده از کمپوست و برداشت محصول در نظر گرفته شود.

Title: بررسی پایایی و روایی مقیاس استنباط فرد از میزان تلاش بر اساس تغییرات ضربان قلب

http://hsr.mui.ac.ir/index.php/jhsr/article/view/1508

مقدمه: یکی از روش‌های برآورد شدت فعالیت، استفاده از مقیاس شدت تلاشدرک شده (Rating of Perceived Exertion)می‌باشد. این مطالعه با هدفبررسی روایی و پایایی نسخه فارسی مقیاس شدت تلاش درک شده بر اساس تغییرات ضربانقلب در دانشجویان دانشگاه علوم پزشكي اصفهان انجام گرفت. روش‌ها: این پژوهش از نوع مداخله‌ای است که بر روی 10 دانشجوی پسرانجام شد، بدین صورت که در دو نوبت در 4 سطح فعالیت سبک، متوسط، سنگین و بسیار سنگینبا استفاده از دستگاه تردمیل در اتاقک شرایط جوی (WBGT=19.4±0.3)، شدت فعالیت افراد در دو مقیاس 10-0و 20-6  شدت تلاش درک شده و تعداد ضربان قلباندازه‌گیری شد. تجزیه و تحلیل داده‌ها با استفاده از آزمون همبستگی پیرسون وآزمون تی زوجی انجام شد. یافته‌ها: ضریب همبستگی بین دو مقیاس 10 و 15 امتیازی شدت تلاش درک شده  با ضربان قلب به ترتیب 83/. و 85/. بود (001/0P<).همچنین ضریب همبستگی بین دو مقیاس 10 و 15 امتیازی شدت تلاش درک شده با ضربان قلبکار به ترتیب 89/. و 91/. به دست آمد (001/0P<). مقادیر دو مقیاس  10 و 15 امتیازی شدت تلاش درک شده در سطوح مختلففعالیت در دو نوبت آزمون اختلاف معنی‌داری نداشتند. نتیجه‌گیری: بر اساس نتایج این مطالعه، هر دو مقیاس 10 و 15 امتیازی شدت تلاش درک شده برایبرآورد شدت فعالیت جسمانی از روایی مناسبی برخوردار می‌باشند.

Title: تاثیر آلاینده‌های هوا بر بیماران قلبی- تنفسی پذیرش شده در بیمارستان‌های منتخب شیراز

http://hsr.mui.ac.ir/index.php/jhsr/article/view/952

مقدمه: پدیده آلودگی هوا از مهمترین مشکلات زیستمحیطی می‌باشد که سلامت انسان‌ها را تهدید می‌نماید. گسترش شهرنشيني و توسعه فعاليت‌هايصنعتي به شدت آلودگي هوا را افزايش داده است كه از عواقب آن تشديد بيماري‌هاي قلبيو تنفسي مي‌باشد. هدف از انجام اين تحقيق بررسي ارتباط بين آلاينده‌هاي هوا وتعداد مراجعه‌کنندگان بیماران قلبی- تنفسی به بیمارستان‌های منتخب آموزشی شیراز است.روش‌ها: این مطالعه از نوعتوصیفی- تحلیلی است. در این مطالعه ارتباط بین آلاینده‌های مونواکسیدکربن (CO)، دی‌اکسیدگوگرد (SO2)،ازن (O3)، و مواد معلق با قطر کمتر از 10 میکرون (PM10) با تعداد مراجعه‌کنندگان بیماران قلبی- تنفسی به بیمارستان‌هایآموزشی شیراز در طی یک دوره آماری شش ماهه ۱۳۹۱-۱۳۹۰ است. سپس با استفادهاز نرم‌افزار آماری SPSS نسخه 16 و با استفاده از روابط رگرسیونپواسنی وآزمون آماری تی به تجزیه و تحلیل تاثیر آلاینده‌های هوا برتعداد مراجعه‌کنندگان به بیمارستان‌های آموزشی شیراز پرداخته شد . سطح معنی‌داریبرابر با 95% اطمینان و 05/0=αاست.یافته‌ها: اگر چه مقایسه میانگین غلظت مونواکسیدکربن،دیاکسیدگوگرد وازن در شهر شیراز با میزان استاندارد نشان داد که میزان آنها در حد استاندارد است، ولیکن میانگین غلظت مواد معلق با قطر کمتر از 10 میکرون  ازحد استاندارد بیشتر می باشد. نتایج آنالیز تک متغیره نشان داد که اگر چه رابطه معنیداری بین مونو اکسید کربن و تعداد مراجعین به بیمارستانهای منتخب وجود دارد(۰/۰5> P) ، ولیکن رابطه بین سایرآلاینده های هوا و تعداد مراجعین دیده نشد (۰/۰5P>).  نتیجه‌گیری: اگر چه مطالعه حاضرنشان داد که مواد معلق با قطر کمتر از 10 میکرون مهمترین عامل آلاینده هوا درشیراز است، ولیکن بیشترین ارتباط بیماران قلبی–تنفسی پذیرش شده دربیمارستانهای منتخب شیراز مربوط به آلاینده مونواکسیدکربناست. درحالی که در تهران رابطه معنی داری بین غلظت دی اکسیدگوگرد  و دی اکسید نیتروژن با تعداد پذیرش شده بیماران در اثر حملات آسم وجود دارد.در استرالیا مهمترین عامل آلاینده که باعث افزایش پذیرش بیماران در بیمارستان بهدلیل مشکلات تنفسی، مرتبط به آلاینده های مواد معلق با قطر کمتر از 10 میکرونو ازن است، در حالی که در امریکا و اروپا ازن مهمترین عامل افزایش در مراجعهبیماران به بیمارستان است.

Title: برنامه‌های پیشگیرانه و کنترلی در مدیریت ریسک‌های بالینی

http://hsr.mui.ac.ir/index.php/jhsr/article/view/944

مقدمه: معمولامشکلات مختلفی درزمینه ایمنی بیمار در حین انجام مراقبت‌های بهداشتی اتفاق می‌افتد. این حوادث شاملخطاهای انتقال خون، دارو یا جراحی اشتباه، صدمات حاصل از جراحی، خودکشی‌های قابلپیشگیری، عفونت‌های اکتسابی، زخم‌های بستر و... است. با توجه به این که درصدغیرقابل اغماضی از بیماران در مواجهه با سیستم‌های نظام سلامت خصوص بیمارستان‌هادچار عوارض و صدمات ناشی از ارائه خدمات می‌گردند و برای اینکه سازمان‌هایبهداشتی- درمانی به عنوان مراکز قابل اطمینان تلقی گردند، لازم است یک نظام اثربخشمدیریت ریسک جهت شناسایی ریسک‌های مختلف و چگونگی مقابله و مدیریت کردن هر یک از آنهاطراحی و اجرا گردد. یکی از عناصر مهم در مدیریت ریسک‌ها وجود برنامه‌های سازمانیپیشگیری از آسیب است.این مطالعه به منظور بررسی و تبیین عوامل موثر در تنظیمبرنامه‌های پیشگیری از آسیب در بیمارستان‌ها تنظیم گردیده است.روش‌ها: اینمطالعه یک پژوهش کاربردی چند مرحله‌ای است که به صورت مقطعی انجام شده است.بهمنظور تدوین الگوی مدیریت ریسک‌های بالینی با هدف شناخت عوامل موثر بر پیشگیری از آسیب وریسک های بالینی از مرور و بررسی متون معتبر مدیریت ریسک دربخش سلامت استفاده شد. مدل‌های مذکور به طور دقیق بررسی ودر قالبجداول تطبیقی با یکدیگر مقایسه ومنطبق شدند. وحدود 62متغیر اولیه استخراج شد ودرقالب یک پرسشنامه  با یک تیم 20 نفره متشکلاز پرستاران وپزشکان اعتبار یابی شد .پرسشنامه نهایی با 39متغیر مورد تایید گروه مذکور قرارگرفت.تایید نهایی الگو از طریق توزیع پرسشنامه در بین جامعه مطالعاتی  درقالب نمونه ای 290نفری که با استفاده از روشخوشه ای وتصادفی اانتخاب شده بودند وهمچنین استفاده از روش تحلیل عاملی اکتشافی انجام شد. داده‌ها با استفادهاز نرم‌افزار SPSS نسخه 18 تحلیل شدند.یافته‌ها: عامل برنامه‌هایپیشگیرانه وکنترلی در الگوی مدیریت ریسک‌های بالینی در مجموع 6/13%از واریانس‌هارا تبیین نمود. این عامل در الگوی مدیریت ریسک‌های بالینی با استفاده از روش تحلیلعاملی اکتشافی سؤال‌ها با چرخش واریماکس در 5آیتم بار گذاری شد. بار عاملیمتغیرهای این عامل دردامنه مطلوبی از8/0 تا 5/0 قرار داشت که نشان‌دهنده تناسبمطلوبیت این متغیرها از دیدگاه جامعه مطالعاتی بوده است. بالاترین متغیر در عامل برنامه‌هایپیشگیرانه وکنترلی ریسک‌های بالینی "استقرارمکانیزم های  مشخص برای شناسایی ریسک از قبیل گزارشحوادث،موارد ارجاعی از طرف پرسنل،مرور مدارک پزشکی بیماران،مرورشکایات بیماران یا بررسیاطلاعات و داده‌های مربوط به بهبود کیفیت " با بار عاملی 818/0 و پایین‌ترینمتغیر "ایجاد و برقراریارتباط موثر با واحدهای کلیدی همچون مدیریت کیفیت، واحد پرستاری، کادر پزشکی و واحدکنترل عفونت به منظور تقوبت تاثیر برنامه مدیریت ریسک " با بار عاملی53/0بود.نتیجه‌گیری: برایاستقرار الگوی مدیریت ریسک‌های بالینی لازم است در کنار سایر عوامل زیرساختی و سازمانینسبت به تهیه تدوین برنامه‌های پیشگیرانه و کنترلی در جهت حذف و کاهش ریسک‌هایموجود در فضای ارایه مراقبت‌های سلامت اقدام شود. این عامل دارای آیتم‌های مختلفیاست که باید در تنظیم و اجرای برنامه مدنظر قرار گیرد. یکی از مهمترین این آیتم‌هاتوجه به آمار و اطلاعات ریسک‌ها و تهیه گزارشات آماری به طور سیستماتیک می‌باشد.

Title: عوامل موثر بر رفتار شیردهی مبتنی بر سازه‌های تئوری اعتقاد سلامتی در زنان سه ماهه سوم بارداری

http://hsr.mui.ac.ir/index.php/jhsr/article/view/1102

مقدمه: علي‌رغم اهمیت تغذيه انحصاری با شير مادر به عنوان يك راهبرد اساسي درتامين رشد و بقاي كودك، ميزان تغذيه انحصاري با شير مادر در ایران با توصيه‌هايسازمان جهاني بهداشت و يونيسف فاصله دارد. در اين راستا، عوامل موثر بر رفتارشیردهی مبتنی بر سازه‌های تئوری اعتقاد سلامتی در زنان سه ماهه سوم بارداری مراجعه‌کنندهبه مراکز بهداشتی درمانی تابعه دانشگاه علوم پزشکی تهران در سال 1391 مورد بررسیقرار گرفت.روش‌ها:اينپژوهش يك مطالعه مقطعی است كه266 نفر از زنان سه ماهه سوم بارداری مراجعه‌کننده بهمراکز بهداشتی درمانی به روش تصادفی در دسترس انتخاب شدند. ابزار گردآوری اطلاعات،پرسشنامه دو بخشی اطلاعات دموگرافیک (8 سؤال) وابزار سنجش رفتار شیردهی بر اساسسازه‌های تئوری اعتقاد سلامت (55 سؤال)بود. داده‌ها بااستفاده از نرم‌افزار SPSS نسخه 18 و آزمون‌های t-test،One- way ANOVA و ضریب همبستگی پیرسون مورد تجزیه و تحلیل قرار گرفت. یافته‌ها:میانگین سن شرکت‌کنندگان 09/4±85/28 سالبود. میانگین نمره منافع درک‌شده در بین سازه‌های تئوری اعتقاد سلامتی بالاترین(93/11±55/81) و خود کارآمدی درک شده (19/5±41/33) پایین‌ترین امتیاز را کسبنمودند. سازه حساسیت درک‌شده با متغیرهای سن(05/0p=) و موانع درک‌شده بامتغیرهای شغل (02/0)، سن (01/0P=) و راهنما برای عمل با سطح تحصیلات زنان باردار (007/0P=)، نمایه توده بدنی (01/0P=) و سن (001/0P=)ارتباط آماری معنی‌داری داشتند. ارتباط آماری معنی‌داری بین سازهمنافع درک‌شده و سطح تحصیلات همسر(024/0P=) مشاهده شد.نتیجه‌گیری: نتایج مطالعه نشان داد که خودکارآمدی درک‌شده درارتباط با رفتار شیردهی در زنان باردار ضعیف می‌باشد. به نظر می‌رسد مداخلات تئوریمحور آموزش بهداشت و ارتقا سلامت مبتنی بر منابع درک شده خودکارآمدی در ارتقارفتار شیردهی موثر باشد.

Title: بررسی اثربخشی آموزش روان درمانی مبتنی بر کیفیت زندگی بر کمال گرایی مراجعان مراکز مشاوره شهر اصفهان

http://hsr.mui.ac.ir/index.php/jhsr/article/view/1192

مقدمه: كمال‌گرايي ويژگي شخصيتي است كه با تلاش براي بي عيب بودنو وضع استانداردهاي بسيار بالا براي عملكردو گرايش به ارزيابي منتقدانه رفتار خود و ديگران مشخص مي‌شود. هدف پژوهش حاضربررسي تاثير كيفيت زندگي درماني بر كمال‌گرايي بوده است.روش‌ها: پژوهش حاضر نیمه تجربي از نوع پيش‌آزمون، پس‌آزمون با گروهگواه بوده، جامعه آماري اين پژوهش افراد مراجعه‌کننده به 3 مراکز مشاوره شهراصفهان (مرکز مشاوره دولتی دانشگاه صنعتی، مرکز مشاوره بوستان، مرکز مشاوره الفبایزندگی) در سال 1391 بودند. نمونه پژوهش شامل 32 مراجعه‌کننده بود که از بين افراد دردسترس انتخاب شده و در دو گروه آزمايش و کنترل قرار داده شدند. مراجعه‌کنندگانگروه آزمايش، به مدت 8 جلسه در هشت هفته تحت آموزش به شيوه کيفيت زندگي درمانيقرار گرفتند و در پايان از هر دو گروه پس آزمون به عمل آمد. ابزاراندازه‌گيري، سياهه كمال‌گرايي هيل با ضريب آلفاي كرونباخ 92/0 است. داده‌ها توسطنرم‌افزار SPSSنسخه 18 و روش آماري تحليل کواريانس مورد تجزيه و تحليل قرار گرفت و05/0P<از نظر آماری معنادار تلقی شد.يافته‌ها:اين تحقيقنشان داد كه قبل از آموزش، اختلاف معني دار بين گروه هاي مورد مطالعه در نمراتكمال گرايي وجود نداشت(P>0/01). اما پس از مداخله آموزشي، افزايشمعني داري در ميانگين نمرات كمال گرايي در گروه مداخله ملاحظه گرديد (01/0P<). همچنيناين روش پس از مداخله آموزشي، بر روي گروه مداخله بر خرده مقياسهاي كمال‌گرايي ازجمله حساسيت بين فردي، تلاش براي عالي بودن، درك فشار از سوي والدين، نظموسازماندهي، هدفمندي ، استانداردهاي بالا براي ديگران وكمال گرايي انطباقي وغيرانطباقي تاثير داشته است(05/0P<).نتيجه‌گيري: نتايج اين پژوهش تأييدکننده تأثير مثبت آموزش کيفيت زندگيدرماني بر کمال‌گرایی افراد مي‌باشد و می‌توان در مراکز درمانی با توجه به ارتباطبیماری‌های روان تنی با کمال‌گرایی و به ویژه در مراکز آموزشی جهت کاهش کمال‌گراییناسالم افراد برنامه‌های عملی تدوین و اجرا نمود.
